# An unusual diagnosis for an usual test

**DOI:** 10.1186/s13052-020-00846-z

**Published:** 2020-06-10

**Authors:** Andrea Trombetta, Vanessa Migliarino, Flavio Faletra, Egidio Barbi, Gianluca Tornese

**Affiliations:** 1 University of Trieste, Trieste, Italy; 2 Institute for Maternal and Child Health “IRCCS Burlo Garofolo”, Trieste, Italy

**Keywords:** Skeletal dysplasia, Hereditary multiple osteochondromas, Growth delay

## Abstract

**Background:**

Hereditary multiple osteochondromas (HMO) is a genetic condition characterized by the presence of multiple osteochondromas, usually at the lateral side of the most active growth plate of a long bone. These lesions may persist, be asymptomatic during childhood, and may increase in number and size until growth plates close. Therefore, diagnosis of HMO in children and young people can be challenging; while short stature can be more evident at the onset of puberty, asymptomatic ostheocondromas can progress into different degrees of orthopedic deformity. Moreover, multiple complications may arise due to the presence of osteochondromas, including tendon and compression muscle pain, neurovascular disorders, obstetric problems, scoliosis and malignant transformation into secondary peripheral chondrosarcoma in adulthood.

**Case presentation:**

We report the case of a girl admitted to our Institute for growth delay. While laboratory tests, including growth hormone stimulation test, were normal, left hand X-ray revealed multiple osteochondromas, suggestive for HMO. The genetic test for *EXT1* and *EXT2* genes confirmed the radiological diagnosis, with a mutation inherited from the mother who displayed the same radiological abnormalities along with recurrent limb pain episodes.

**Conclusions:**

HMO is a genetic condition whose diagnosis can be challenging, especially in females. Every pediatricians should consider a skeletal dysplasia in case of unexplained growth delay and a skeletal survey might be fundamental in reaching a diagnosis.

## Background

HMO is an autosomal dominant disorder characterized by the presence of multiple osteochondromas, usually at the lateral side of metaphyses of long bones. The prevalence of such condition is reported to be 1 in 100.000, although the real incidence could be higher because mild phenotypes are sometimes not detected [1, 2]. Two genes, *EXT1* (OMIM: 608177) and *EXT2* (OMIM: 608210), which are located respectively at 8q24.11 and 11p11.2, have been identified to cause HMO [3, 4], with an estimated penetrance of 100% [5] and variable expressivity, especially in females [2]. Mutations of such genes lead to an insufficient elongation of heparan sulfate chains and therefore contribute to disrupt chondrocyte differentiation and proliferation pathways [6].

Osteochondromas may persist, be asymptomatic during childhood, and may increase in number and size until growth plates close. Whereas this condition is generally diagnosed before the age of 10 [7], sometimes the diagnosis of HMO in children and young people can be challenging; while short stature can be more evident at the onset of puberty, asymptomatic ostheocondromas can progress into different degrees of orthopedic deformity. On the other hand, early diagnosis is essential for the follow-up of potential complication of such condition. In fact, multiple complications may arise due to the presence of osteochondromas, including tendon and compression muscle pain, neurovascular disorders, obstetric problems, scoliosis and malignant transformation into secondary peripheral chondrosarcoma in adulthood. On the other hand, HMO can affect quality of life and global health status, especially in female patients [8].

## Case presentation

A 13-year-old girl was referred to our Institute for growth delay. Her medical history was unremarkable, and she was asymptomatic. She was born at term, with adequate weight (3080 g, − 0.51 SD) and length (49 cm, − 0.51 SD).

At admission, her height was 146.8 cm (− 1.37 SD), weight 36 kg (− 1.71 SD), body mass index (BMI) 16.71 kg/m2 (− 1.39 SD), pubertal stage Tanner 3, with a growth velocity of 5.8 cm/year (− 2.11 DS). The mid parental height was 175 cm (1.69 SD) with no short stature in both parents.

Physical examination was unremarkable, with regular sitting height/height ratio (0.51, 0 SD), without pterygium, cubitus valgus, or forearm malformations.

**Fig. 1:**
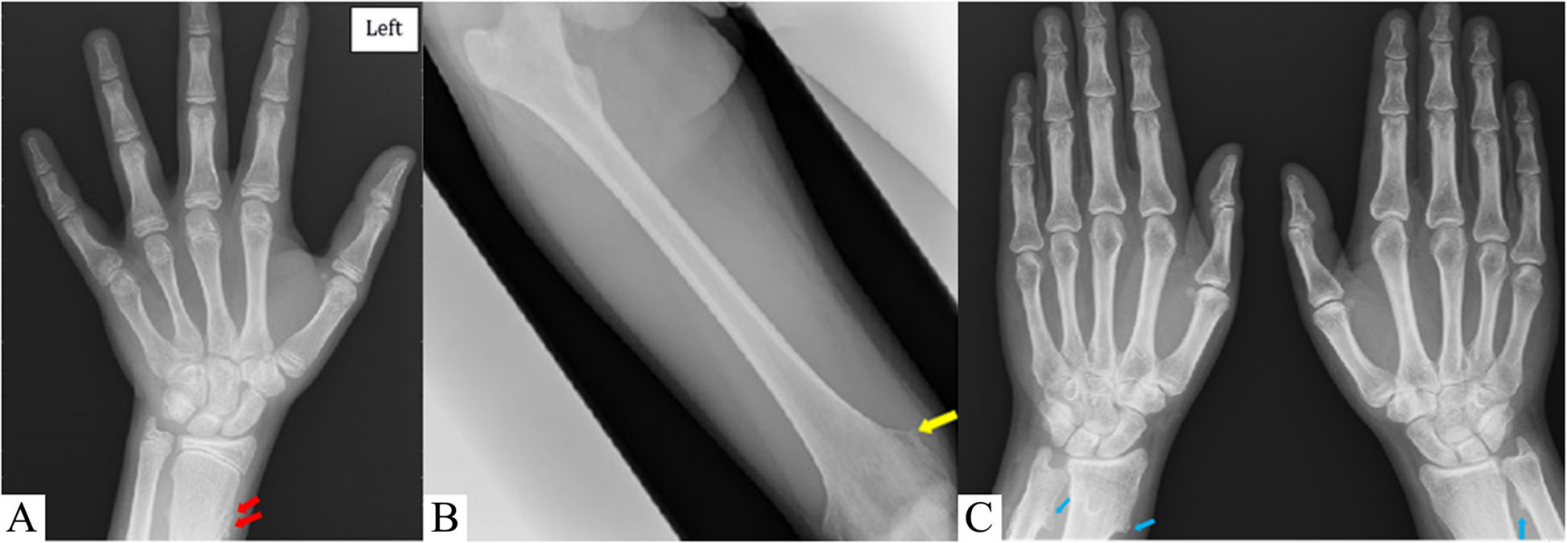
**a** Left hand X-ray, showing multiple bulky cartilage cap in the distal region of the radius suggesting multiple osteochondromas (red arrow). **b** Right femur X-ray, showing an uninterrupted flow of cortex and medullary bone from the host bone into an area of new bone proliferation in the medial side of the distal femur (yellow arrow), confirming the hypothesis of multiple osteochondromas. **c** Bilateral hands X-rays of the girl’s mother, showing multiple osteochondromas (only a few of which outlined by blue arrows)

Due to the marked growth failure, with the height velocity above the 25th percentile for age despite the girl did not exhibited short stature, we suspected a growth hormone deficiency. Therefore, a growth hormone (GH) stimulation test with arginine was performed, with normal values (peak 8.8 ng/ml). Also other common causes of delayed growth, such as hypothyroidism, chronic conditions, malnutrition, Cushing disease, Turner syndrome, celiac disease, were ruled out by laboratory tests. The left-hand X-ray, performed in order to determine the bone age (13 years according to Greulich and Pyle), ruled out GH deficiency because of correspondence of chronological age and bone age. In addiction, such test did not show deformities compatible with *SHOX* gene deficiency or Turner syndrome but revealed the presence of multiple osteochondromas (Fig. 1a). Such lesions were present not only in hand but also at the lateral side of the most active growth plate of a long bone (Fig. 1b) highly suggestive for HMO.

During further investigation in family history, her mother reported a history of recurrent limb pain episodes and X-ray revealed multiple osteochondromas (Fig. 1c). The radiologic and anamnestic hypothesis of HMO was confirmed by the Sanger sequencing analysis of the *EXT1* gene, which showed a c.138_139delinsTCT (p.Leu46Phefs*143) frameshift mutation, not previously described in literature, in the girl and mother.

The patient was further monitored, with the absence of spine radiological anomalies. Her growth velocity slightly improved to 6.2 cm/year, and the girl did not complain about any painful episode in the following year.

## Discussion and conclusions

Hereditary multiple osteochondromas (HMO) is an autosomal dominant skeletal disorder which alters enchondral bone during growth and is characterized by the presence of multiple osteochondromas, usually in growth plates of the long bones that are most active in growth. These lesions may persist, be asymptomatic during childhood, and may increase in number and size until growth plates close [9]. HMO may present with different degrees of orthopedic deformity, and multiple complications may arise due to osteochondromas, including tendon and compression muscle pain, neurovascular disorders, obstetric problems, scoliosis and malignant transformation into secondary peripheral chondrosarcoma in adulthood [9]. A clinical classification system was developed to better characterize HMO, based on the number of bone segments and the presence of skeletal deformities and functional limitations (Table 1) [10].

**Table 1:** Clinical classification of HME

I	No deformities and no functional limitations	A ≤ 5 sites with osteochondromas
		B > 5 sites with osteochondromas
II	Deformities and no functional limitations	A ≤ 5 sites with deformities
		B > 5 sites with deformities
III	Deformities and functional limitations	A Functional limitation of 1 site
		B Functional limitation of > 1 site

A genetic test for *EXT1* and *EXT2* genes, which are responsible for this condition, should be performed in case of detection of multiple osteochondromas. The *EXT1* gene should first be analyzed, taking into account the prevalence of pathogenic variants. The combined analysis of the entire coding regions of both *EXT1* and *EXT2* gene detects pathogenic variants in 70–85% of affected individuals [2]. In case of deletions or duplications which are not identified by the Sanger sequencing method, multiplex ligation-dependent probe amplification (MLPA) analysis can be employed, due to the significant amount of exonic deletion in HMO, with a detection rate reported to be increased to as much as 85–95% when combined to other tecniques [2].

Finally, a genetic test should also be performed in their parents, due to the low rate (10%) of de novo mutations.

Although individuals with HMO sometimes present short stature, more evident after puberty, not only as a consequence of the involvement of long bones growth plates but also for the systemic influence of the gene defect on growth rate [11], our patient did not display pathological short stature along with a pathological growth delay. Height distribution significantly differs during childhood, worsening with age until adulthood [12]. The normal maternal height in our case was an element of diagnostic difficulty and might be explained by the wide clinical expressivity of this condition, depending on the reduction of heparin sulfate and the subsequent impact on a number of signalizing proteins critical to skeletal development, such as IHH, PTHrP, FGF, BMP and WNT4. Furthermore, since the presence of knee osteochondromas, in particular in the distal femur, is an independent predictor of knee deformity and short stature [13], radiographs of the knee can be useful not only to detect mildly affected individuals, but also to predict range of movement limitation. When GH deficiency is associated, these patients may benefit from replacement therapy to improve their final height. Despite the effect of GH on the growth plate and chondrocyte proliferation, no substantial evidence on osteochondromas development has been reported. The follow-up of lesions is mandatory during the replacement therapy since the most frightening complication is the transformation into a malignant chondrosarcoma [14]. Along with clinical monitoring, a periodic assessment with full spinal MRI should be performed in order to rule out the intraspinal osteochondromas and possible neurological damage [15].

In adulthood, a periodic clinical examination should be performed every 12–24 months to detect the early signs of a malignant transformation in particular in high-risk areas such as pelvis, scapula, and proximal femur. Treatment is generally conservative and directed at pain management since lesions can regress during childhood and adolescence [16], although skeletal deformities or painful osteochondromas should be surgically treated. Surgery should be considered in case of large and symptomatic osteochondromas or addressed to deformities [17].

Skeletal dysplasia is often underdiagnosed, even in the context of short stature: in a large cohort of children with idiopathic short stature, after a skeletal survey, 21.8% were found to have an undiagnosed skeletal dysplasia [18]. Reaching a diagnosis can ensure orthopaedic surveillance and specific treatment in appropriate situations.

Besides an accurate history (including family history, joint pain and ligamentous laxity or joint contracture) and a meticulous physical examination (including upper/lower segment ratio measurement and minor dysmorphism) [19], a skeletal survey may be indicated in any case of otherwise undiagnosed growth delay and short stature, starting from a careful evaluation of the ordinarily performed left-hand X-ray (which can be sufficient as in this case or in *SHOX* gene deficiency) down to a complete skeletal survey.

Therefore, every paediatrician should consider a skeletal dysplasia in case of unexplained growth delay and a cooperative approach by clinicians, radiologists and geneticists is essential to reach an exact diagnosis.

## Data Availability

Not applicable.

## Abbreviations

HMO: Hereditary multiple osteochondrosis
SD: Standard deviations
BMI: Body mass index;
GH: Growth hormone
MRI: Magnetic resonance imaging
